# Effects of a CB_2_ Subtype Selective Agonist ABK5-1 on Cytokine Production in Microglia

**DOI:** 10.33696/signaling.2.038

**Published:** 2021

**Authors:** Yaliang Tang, Barbara Wolk, Debra A. Kendall

**Affiliations:** Department of Pharmaceutical Sciences, University of Connecticut, Storrs, Connecticut 06269, USA

**Keywords:** CB_2_ receptor, Cytokines, Chemokines, Inflammation, Microglia

## Abstract

**Background and objectives:**

Neuroinflammation is closely associated with various diseases including neuropathic pain. Microglia are immune cells in the central nervous system which are the main players of immunity and inflammation. Since microglia are activated by nerve injury, and they produce proinflammatory mediators to cause neuropathic pain, targeting activated microglia is considered to be a strategy for treating neuropathic pain. Activation of the cannabinoid CB_2_ receptor is known to have anti-inflammatory effects in microglia. ABK5-1 is a CB_2_ subtype selective agonist which inhibits IL-1β and IL-6 production in the microglia cell line BV-2. The purpose of the current study is to further analyze anti-inflammatory effects of ABK5 in terms of different cytokines and the possible pathway involved in the effect in the BV-2 cell line.

**Methods:**

A cytokine array was performed to screen the effect of ABK5-1 on forty inflammatory mediators in BV-2 cells. Changes of the inflammatory mediators was further supported by mRNA analysis, and a possible signaling molecule that involved the observation was evaluated by western blot.

**Results:**

Stimulating BV-2 cells by lipopolysaccharide increased expression of eleven inflammatory mediators, and ABK5-1 treatment resulted in more than a 50% decrease of sICAM1, IL-6, and RANTES. Real-time PCR results showed a decrease of G-CSF, ICAM1, MCP-1, MIP-1α, and MIP-1β mRNA levels. Western blot analysis showed that ABK5-1 inhibited LPS-induced ERK phosphorylation, which can be a mechanism of ABK5-1-mediated anti-inflammatory effect.

**Conclusions:**

Our current results support the possibility that ABK5-1 is an anti-inflammatory drug for microglia.

## Introduction

Cannabis has been used for centuries for medical reasons and is known to have various beneficial impact such as anti-inflammation and analgesic effects [1], while it may also cause some unwanted effects. Δ^9^-tetrahydrocannabinol (THC) is a well-studied component of cannabis which is known to cause these effects by binding both subtypes of cannabinoid receptors, CB_1_ and CB_2_. Cannabinoid receptors are GPCRs which primarily couple to Gi/o protein. Activation of cannabinoid receptors typically decreases cellular cAMP concentration (through coupling to Gi proteins), modulates the activity of ion channels, and induces signal transduction through various signaling pathways including ERK [2]. Since CB_1_ is mainly expressed in the central nervous system (CNS) and involved in control of neurotransmitter release [3,4]. While it expected to be a good target for some diseases, unwanted psychoactive side effects such as euphoria, dizziness, and impairment of memory are considered be associated with CB_1_ activation [5]. In contrast, although CB_2_ also can be found in some populations of cells in the CNS, its expression is mostly in immune cells and peripheral tissues [6,7], and activation of CB_2_, is reported to have anti-inflammation effects and analgesic effects without the psychoactivity [8-11]. Therefore, selectively targeting CB_2_, has been a good strategy for obtaining new drugs to treat inflammation and pain.

Microglia are macrophage-like cells in the CNS which play important role in immunity and homeostasis of the CNS. As immune cells, microglia are activated by pathogens and damaged cells, and remove the pathogens and dead cells through phagocytosis and production of inflammatory cytokines [12]. However, excessive and prolonged pro-inflammatory cytokine production by activated microglia causes various diseases including chronic pain [12-14]. Activation of microglia is known to be associated with neuropathic pain, which is one of major types of chronic pain [13,14]. Neuropathic pain is caused by nerve injury, and the damaged nerve releases mediators such as ATP, MMP-9 and other chemokines, which activate microglia [15]. Activated microglia releases pro-inflammatory cytokines including tissue necrosis factor (TNF)-α, interleukin (IL)-1β, and IL-6 [13,15]. These cytokines bind to their receptors in pre-synaptic and post-synaptic neurons and change expression and activity of receptors of neurotransmitters and ion channels [13], and result in change of excitability of neurons. These changes lead to allodynia and hyperalgesia, which are typical symptoms of neuropathic pain [16-18].

**Figure 1:**
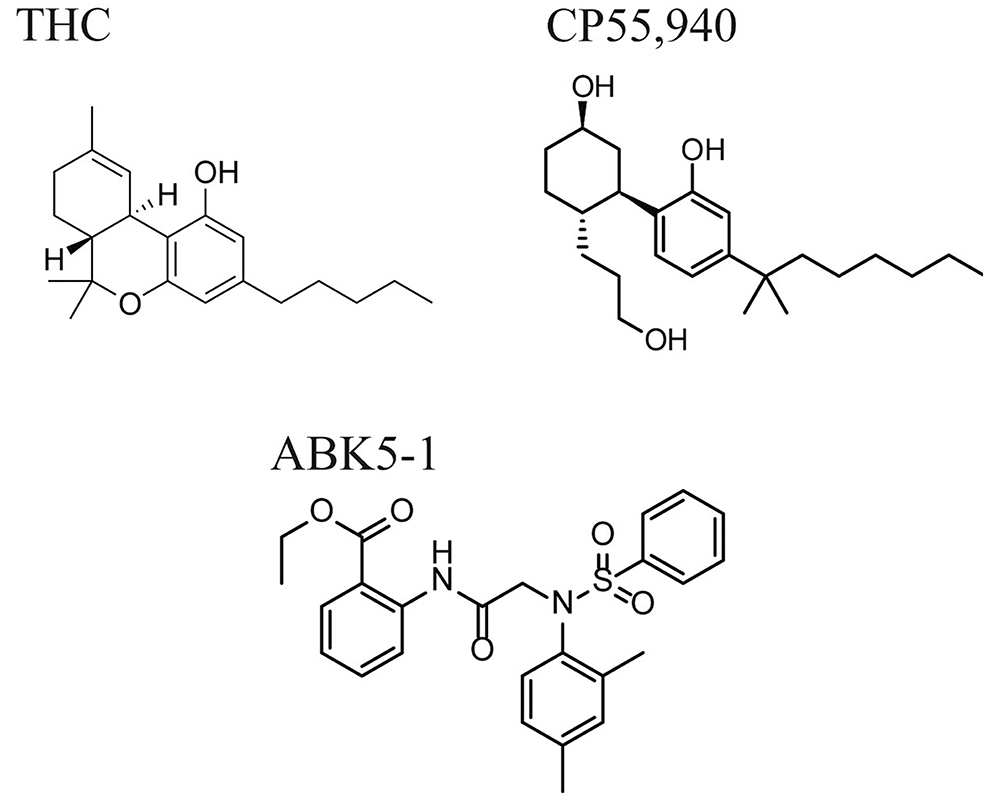
Chemical structures of cannabinoid receptor agonists and ABK5-1. THC and CP55,940 are non-selective cannabinoid receptor agonists which bind both CB_1_ and CB_2_. ABK5-1 is a CB_2_ subtype-selective agonist.

CB_2_ receptors are expressed in immune cells including microglia, and activation by CB_2_, agonists can reduce pro-inflammatory cytokine production in those cells [19-26]. Previously, we have reported that a CB_2_ subtype-selective agonist, ABK5, which is a compound with a distinct structure from the known CB_2_, agonists, has anti-inflammatory effects in T-cells. ABK5 inhibited IL-2 and TNF-α production and chemotaxis induced by CXCL12 [27,28]. To optimize ABK5 for better efficacy, we introduced several analogs of ABK5, and found ABK5-1 (Figure 1) had a good binding affinity to CB_2_ (K_i_=28 nM) without CB_1_ binding. Similar to its lead compound, ABK5-1 also had anti-inflammatory effects, not only in T-cells but also in the microglial cell line BV-2. In this study, to further evaluate the impact of ABK5-1 on cytokine production in microglia, we globally analyzed the changes in the levels of many cytokines. In addition, the effect of ABK5-1 on some components of signal transduction was also analyzed as a possible mechanism of the anti-inflammatory effects.

## Materials and Methods

### Reagents

ABK5-1 was purchased from ChemBridge Corporation (San Diego, CA USA). Lipopolysaccharide (LPS) was obtained from Millipore Sigma (St. Louis, MO USA). Phospho-p44/42 MAPK (pERK) and p44/42 (ERK) rabbit monoclonal antibodies were obtained from Cell Signaling Technology (Danvers, MA USA). IRDye 680RD Goat anti-Rabbit IgG (H + L) was purchased from LI-COR Biotechnology (Lincoln, NE USA).

### Cell culture and drug treatment

BV-2 cells were cultured in Minimum Essential Medium (Life Technologies, Carlsbad, CA USA) supplemented with 10% fetal bovine serum and maintained at 37°C and 5% CO_2_ saturation.

For ABK5-1 treatment, BV-2 cells were seeded at 1 × 10^5^ cells/mL and allowed to grow for 24 hours. After 24 hours, cells were treated with 10 μM ABK5-1 or vehicle (DMSO) for 30 minutes in serum free Minimum Essential Medium and then stimulated with 1 μg/mL LPS for 24 hours. 500 μL of cell culture supernatant was collected and subjected to cytokine arrays using Proteome Profiler Mouse Cytokine Array Kit, Panel A (R&D systems, Minneapolis, MN USA) following the manufacturer instructions. The signal was detected by a ChemiDoc MP Imaging System (Bio-Rad Laboratories, Hercules, CA USA). Cells were collected for western blot analysis or mRNA analysis by real-time PCR.

### Quantitative real-time PCR

BV-2 cells were lysed and total RNA was extracted using TRIzol Reagent (Life Technologies, Carlsbad, CA USA) followed by reverse transcription by a High-Capacity cDNA Reverse Transcription kit (Life Technologies, Carlsbad, CA USA) according to the manufacturer’s instructions. Quantitative real-time PCR was performed using an Applied Biosystems 7500 Fast Real-Time PCR system in a 10 μL reaction volume containing 2 μL diluted cDNA and 0.5 μM each of forward and reverse primers, and PowerUp SYBR Green Master Mix (Life Technologies, Carlsbad, CA USA). Primers used in the reaction are as follows: mouse 36B4 forward, 5’-ACTGGTCTAGGACCCGAGAAG-3’, and mouse 36B4 reverse, 5’-TCCCACCTTGTCTCCAGTCT-3’, and mouse CSF3 forward, 5’-CCCGAAGCTTCCTGCTTA-3’, and mouse CSF3 reverse, 5’-GGGGTGACACAGCTTGTAGG-3’, and mouse ICAM-1 forward, 5’-TTTGGGATGGT AGCTGGAAG-3’,mouse ICAM-treverse,5’-TTTGGGATGG TAGCTGGAAG-3’, and mouse MCP-1 forward, 5’-CA TCCACGTGTTGGCTCA-3’,mouse MCP-1 reverse,5’-GCTG CTGGTGATCCTCTTGT-3’, and mouse MIP-1a forward, 5’-TGCCCTTGCTGTTCTTCTCT-3’, mouse MIP-1a reverse, 5’-GATGAATTGGCGTGGAATCT-3’, and mouse RANTES forward, 5’-TGCAGAGGACTCTGAGACAGC-3’, mouse RANTES reverse, 5’-CCATATGGTGAGGCAGGTG-3’. The PCR cycles are as follows: 50°C for 2 min, 95°C for 2 min, 40 cycles of 95°C for 3 sec and 6o°C for 30 sec.

### Western blotting

BV-2 cells were harvested and lysed in lysis buffer 6 (R&D systems, Minneapolis, MN USA) supplemented with protease inhibitor cocktail (MilliporeSigma, Burlington, MA USA) on ice for 30 minutes. The cell lysates were then treated with β-mercaptoethanol and 15 μg of protein was loaded to each well of polyacrylamide gels, and subjected to electrophoresis. Proteins in the gels were transferred to polyvinylidene fluoride (PVDF) membrane (MilliporeSigma; Burlington, MA USA) and blocked with Superblock T20 (PBS) blocking reagent (Fisher Scientific; Pittsburgh, PA, USA) overnight, followed by incubation with primary antibodies (pERK and ERK) for 2 hr and secondary antibodies (IRDye goat anti-rabbit IgG) for 1 hr at room temperature. Bands were detected by the Odyssey CLx imaging system (LI-COR Biotechnology, Lincoln, NE USA).

### Data analysis

Results are presented as the mean ± standard error of the mean (S.E.M.). Signals of dot blots were quantified by pixel density using ImageJ. Western blot bands were quantified by intensity of fluorescence by Image Studio (LI-COR Biotechnology, Lincoln, NE USA). One-way ANOVA followed by Dunnett’s post hoc test was used to determine significant differences from the control group.

## Results

### Cytokine screening in microglia

**Figure 2:**
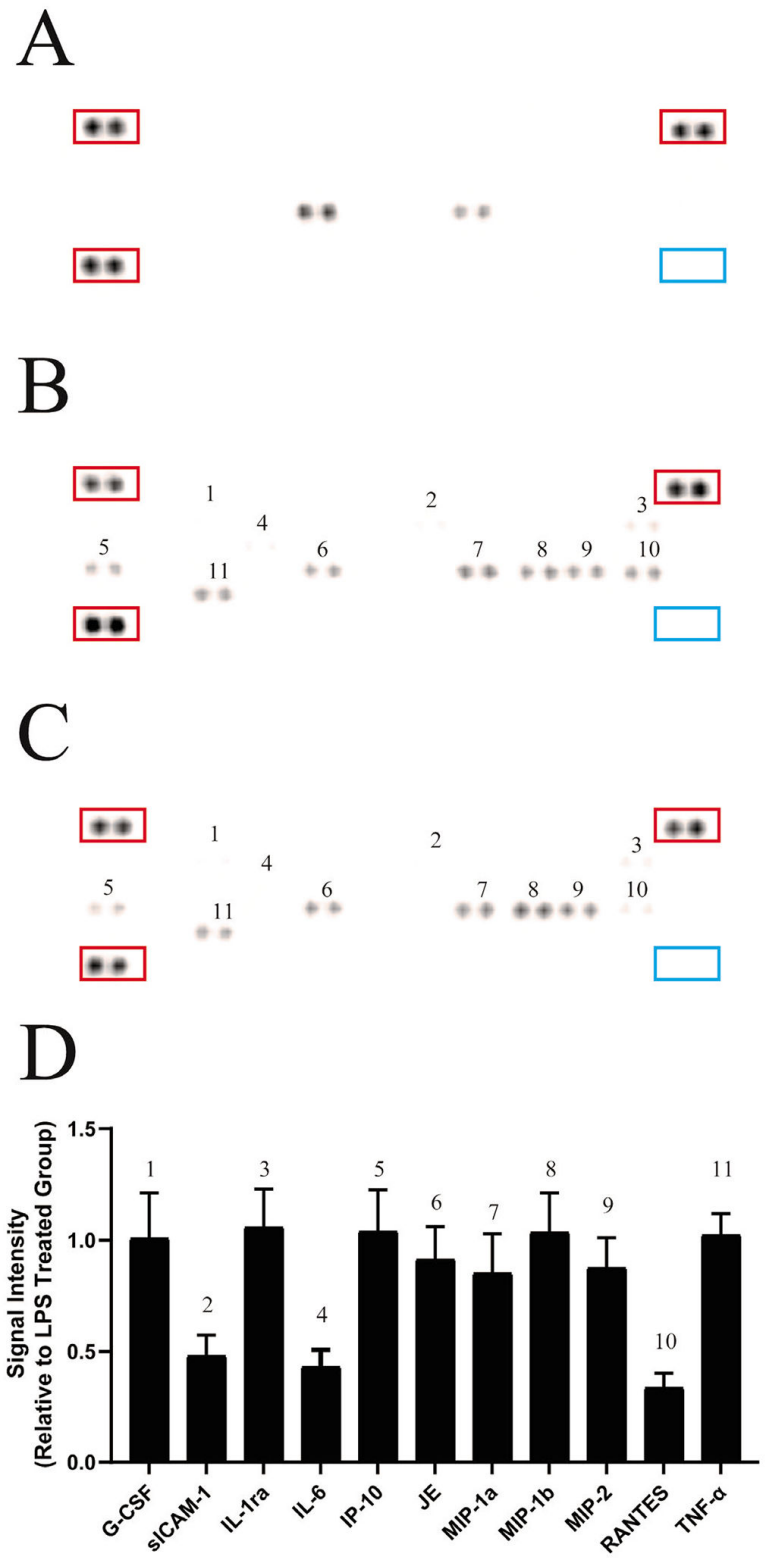
Cytokine array analysis using BV-2 culture supernatant. Cytokines released from **(A)** vehicle-treated cells, **(B)** cells stimulated with LPS, **(C)** cells treated with both LPS and ABK5-1. The culture medium was analyzed by dot blot using membranes with forty antibodies for cytokines and chemokines immobilized. **(D)** Quantification of the signal intensity of dots. Each column represents the relative intensity of ABK5-1- and LPS-treated cells together to cells treated with LPS alone. The experiment was repeated three times and representative blots are shown. Quantification was performed in three blots for each group (LPS alone and LPS+ABK5-1 treated groups) and an average of 9 dots are shown in the graph. Numbers on or near each pair of dots represent different signals for different proteins and correspond to numbers in the graph. Dots in the red rectangles are positive controls and blue rectangles are negative controls.

Activation of the CB_2_ receptor is known to be associated with a decreased production of various pro-inflammatory cytokines such as IL-1β, IL-6, and TNF-α. [26,29-31]. To best characterize anti-inflammatory effects of our compound ABK5-1, we screened for changes in the levels of various cytokines and chemokines after LPS stimulation and LPS stimulation plus ABK5-1 treatment in BV-2 mouse microglia cell line. Dot blot analysis was performed using membranes with forty antibodies for cytokines and chemokines immobilized and the cell culture supernatant from BV-2 cells was treated with vehicle (Figure 2A), LPS (Figure 1B), and both LPS and ABK5-1 (Figure 1C). Eleven cytokines and chemokines in total were detected after 24 hr stimulation with LPS, and compared with vehicle treated cells, nine were increased by LPS treatment (Figures 2A and 2B). IL-1β, which is known to be induced by LPS stimulation in BV-2 cells was not detected under the current conditions. We then analyzed the effect of ABK5-1 on these eleven cytokines and chemokines which could be detected in LPS treated cells to evaluate if ABK5-1 decreased the cytokine levels increased by LPS stimulation. ABK5-1 treatment in LPS-stimulated cells decreased three total cytokines and chemokines. They are soluble intercellular adhesion molecule 1 (sICAM1), IL-6, and regulated upon activation, normal T cell expressed and pesumably secreted (RANTES) and the level was decreased more than 50% compared with cells stimulated with LPS alone (Figures 2B-2D). In addition, monocyte chemotactic protein 1 (MCP-1), macrophage inflammatory proteins (MIP)-1α, and MIP-2 also showed a decreasing tendency by ABK5-1 treatment. In contrast, although reported to be inhibited by CB_2_ activation, LPS-induced TNF-α in the culture medium did not change by ABK5-1 treatment. ABK5-1 did not affect cell viability (data not shown).

### Effects of ABK5-1 on cytokine mRNA levels

**Figure 3:**
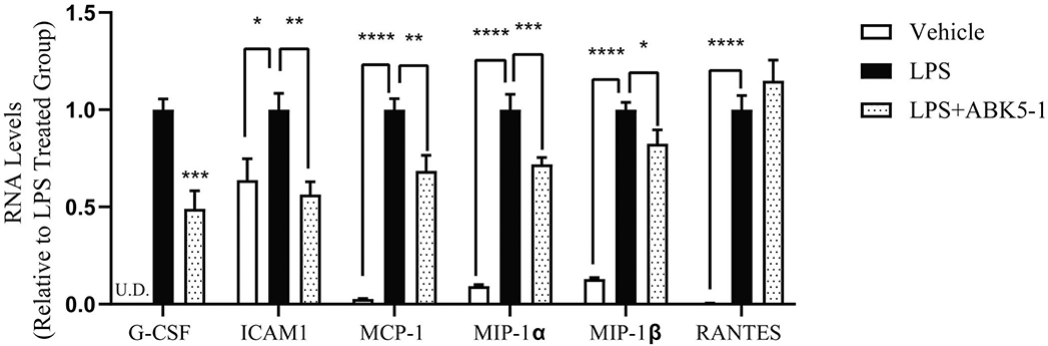
mRNA levels of selected cytokines and chemokines. BV-2 cells were pretreated with 10 μM ABK5-1 or DMSO and then stimulated with LPS or vehicle for 24 hr. Results are presented as relative to cells stimulated with LPS in the absence of ABK5-1. One-way ANOVA plus Dunnett’s post-hoc test were used, and *p<0.05, **p<0.01, ***p< 0.001, ****p<0.0001 (versus LPS alone). U.D. means under the detection limit.

To confirm the change of cytokines by ABK5-1, mRNA levels of selected cytokines and chemokines were evaluated by RT-PCR (Figure 3). All six mRNAs of cytokines and chemokines were induced by 24 hr LPS treatment. Although protein level of Granulocyte colony-stimulating factor (G-CSF) decrease was not observed in cytokine array, ABK5-1 treatment significantly decreased mRNA level by 51% compared with cells treated with LPS alone. The mRNA level of ICAM1, which is the gene of sICAM-1, decreased by 44% by ABK5-1 treatment and this was similar to the level before LPS treatment. Three chemokines, MCP-1, MIP-1α, and MIP-1β mRNA levels also significantly decreased by 31%, 28%, and 17%, respectively. These results support the effect of ABK5-1 on cytokine and chemokine production in BV-2 cells. In contrast, interestingly, mRNA level for RANTES did not change by ABK5-1 treatment despite more than 50% decrease was observed in protein level in cell culture medium (Figure 2D).

### Analysis of ERK phosphorylation

**Figure 4:**
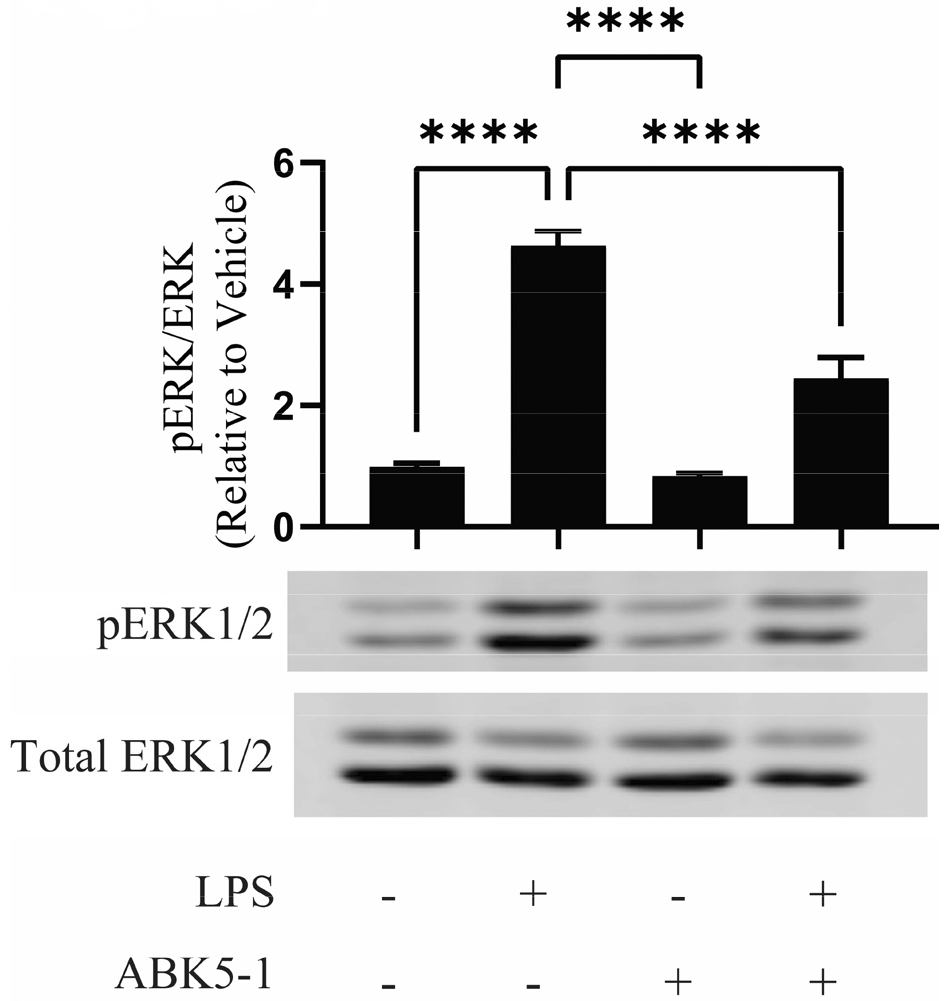
ERK1/2 phosphorylation by LPS and the effect of ABK5-1. BV-2 cells were treated with vehicle (DMSO, <0.1%) or ABK5-1 for 30 min and then stimulated with LPS for 24 hr. Representative blots for pERK1/2 and total ERK1/2 are shown. The experiment was repeated three times and quantification indicates the average of blots from three independent experiments. One-way ANOVA plus Dunnett’s post-hoc test were used, and ****p<0.0001 (versus LPS alone).

LPS binds to the toll-like receptor (TLR) 4 and promotes production of cytokines through a number of different signaling pathways including MAPK pathways. CB_2_ activation by cannabinoid receptor agonists such as anandamide, WIN 55,212-2, and JWH-015 have been reported to inhibit ERK1/2 phosphorylation in microglia and decreased the production of inflammatory mediators [22,24]. To examine if ABK5-1 also has a similar effect, we evaluated ERK1/2 phosphorylation in BV-2 cells treated with LPS, ABK5-1, and both LPS and ABK5-1. After 24 hr of incubation, LPS significantly increased ERK1/2 phosphorylation while ABK5-1 did not change ERK1/2 phosphorylation (Figure 4). Co-incubation of LPS and ABK5-1 resulted in significant decreases of phospho- ERK1/2 level relative to cells treated with LPS alone (Figure 4). This suggests that the effect of ABK5-1 on cytokine production may also be associated with a decrease of ERKt/2 phosphorylation.

## Discussion

Inhibiting neuroinflammation through targeting microglia is considered to be a good strategy for finding new therapeutics for various diseases including neurodegenerative disorders and chronic pain. Chronic pain has been a major health concern because it severely affects people’s quality of life, and also there is a lack of safe and effective drugs for treatment of high-impact chronic pain which may lead to the public health issue (e.g. the opioid crisis). Neuropathic pain is one of the major types of chronic pain, and activation of microglia by inflammatory mediators released from damaged nerves is known to be involved [13,14]. Since activated microglia further produce a variety of inflammatory mediators which change excitability of neurons and cause pain [13,15] reduction of pro-inflammatory cytokine production in microglia is a first step in targeting microglia to reduce pain.

Cannabinoids are known to have anti-inflammatory effects and activation of CB_2_ receptor is considered to be responsible for these effects [32]. In contrast to the CB_1_ receptor which is mainly localized in neurons, the CB_2_ receptor is primarily expressed by immune cells such as macrophages, T cells, and microglia [6,7]. Since selective activation of CB_2_ does not cause psychoactive effects [8,11], CB_2_ subtype selective agonists are good drug candidates for reducing neuroinflammation and associated pain. We have previously reported that a CB_2_ subtype selective agonist ABK5, which has a distinct structure from known cannabinoid receptor agonists, inhibits inflammation in human T cells [28]. ABK5-1 is an ABK5 analog which is also a subtype selective CB_2_ agonist with marginally stronger anti-inflammatory effects than the lead compound. Since this compound also inhibited production of IL-1β and IL-6 in microglia, we were interested in what other inflammatory mediators it can modulate and the possible signaling pathway that was involved in the effects.

LPS stimulation induced nine cytokines and chemokines in BV-2 cells. Induction of IL-6 was observed and the level of IL-6 decreased by ABK5-1 treatment (Figure 2). This was compatible with our previous observation in RT-PCR and ELISA, in which ABK5-1 significantly decreased IL-6 mRNA and protein levels. In contrast, IL-1β, which is a cytokine that is known to be induced by LPS in microglia, was not detected in the cytokine array using cell culture medium. We have previously observed an increase of IL-1β mRNA in BV-2 cells by LPS stimulation, but IL-1β protein concentration in the culture medium was much lower than that of IL-6. Therefore, IL-1β concentration in the culture medium might be under the detection limit of the current method. Cannabinoid receptor agonists have been reported to decrease TNF-α in primary microglia and N9 microglia cell lines [29]. Although TNF-α was induced by LPS in the present study, ABK5-1 treatment did not show an inhibitory effect on TNF-α release to cell culture medium. This can be a limitation of BV-2 cells, or can be also be a compound-dependent observation. Some CB_1_ receptor agonists are known to cause biased signaling in which only certain signaling pathways are activated depending on which agonist is used [33]. Considering the complexity of the signaling pathway downstream of the TLR4 and the CB_2_ receptor, ABK5-1 may only activate certain pathways which are involved in regulation of IL-1β and IL-6, but not TNF-α. It would be interesting to perform a phospho-protein array in primary microglia and compare with compounds which are known to inhibit TNF-α production in the future.

To support the anti-inflammatory effect of ABK5-1 which was evaluated by cytokine array analysis, we also checked mRNA levels of selected cytokines and chemokines in cells stimulated with LPS with and without ABK5-1. Although the change of mRNA levels for most genes were compatible with the change in protein levels observed in the cytokine array analysis, G-CSF and RANTES were the exceptions. G-CSF only decreased in the mRNA level but not in the protein level, while RANTES only changed in the protein level but not the mRNA level. Since mRNA changes are faster than changes in the protein level, doing time course analysis might be helpful to further analyze the change of these two inflammatory mediators. In addition, ABK5-1 may also change the expression of these proteins through modulating post-transcriptional regulation. Further study is needed to clarify the mechanism of this difference between mRNA and protein levels.

Although some studies showed an increase of antiinflammatory cytokines such as IL-4 and IL-10 after activation of CB_2_ by various methods [26,29,34-36], we did not observe increase of such cytokines in the current condition. This might be due to the difference between different cell lines and primary microglia and the difference of the stimulation method. However, we cannot exclude the possibility that a lack of effect on anti-inflammatory cytokines is compound dependent and our compound does not cause an increase of anti-inflammatory cytokines, since certain compounds seem to increase IL-10 by CB_2_ agonist in BV-2 cells [36]. Considering anti-inflammatory cytokines are produced by a different population of microglia which have undergone alternative activation, it will be interesting to compare the change of microglial phenotype between known CB_2_ agonists and ABK5-1.

The endocannabinoid, anandamide, and some other cannabinoid receptor agonists are known to inhibit LPS-mediated ERK1/2 phosphorylation and consequent transcription of inflammatory mediators in microglia [22,24]. Consistent with these findings, ABK5-1 also inhibited LPS-induced ERK1/2 phosphorylation in BV-2 cells. This change may also be associated with a decrease of cytokine by ABK5-1. However, there are more signaling molecules, such as p38 and Akt pathways, which may also play roles in LPS-mediated cytokine production. Since cannabinoid receptor activation also modulates these pathways, ERK1/2 may not be the only mechanism for the anti-inflammatory effects of ABK5-1. Future studies will include broader evaluation of signaling pathways involved in anti-inflammation and the effect of ABK5-1 on it.

In conclusion, we have analyzed LPS-induced cytokines and chemokines inhibited by the CB_2_ subtype selective agonist ABK5-1, and found three cytokines and chemokines were affected by the compound. Change of the inflammatory mediators were also supported by measuring individual mRNA levels. This may be associated with inhibition of ERK1/2 phosphorylation. These observations support that ABK5-1 has anti-neuroinflammation effects.

## Future Research Directions

Since the present study was mainly conducted in BV-2 cells, which may not completely reflect reactions of microglia in the body, it will be interesting to evaluate cytokine production (especially anti-inflammatory cytokines) and activation markers after ABK5-1 treatment in primary microglia isolated from rodents. Testing if ABK5-1 can pass the blood-brain barrier will be essential before performing animal studies to determine if the compound can be effective in neuropathic pain model animals. Finally, performing a behavioral test to evaluate if the compound can cause psychoactive effects in rodents and testing the analgesic effects in neuropathic pain models will provide strong evidence that this compound is a good drug candidate for treating neuropathic pain.

## Abbreviations

CNS: Central Nervous System
IL: Interleukin
LPS: Lipopolysaccharide
G-CSF: Granulocyte Colony-stimulating Factor
RANTES: Regulated upon Activation, Normal T cell Expressed and presumably Secreted
sICAM: soluble Intercellular Adhesion Molecule 1
MCP-1: Monocyte Chemotactic Protein 1
MIP: Macrophage Inflammatory Proteins
THC: Δ^9^- Tetrahydrocannabinol
TLR: Toll-like Receptor
TNF: Tissue Necrosis Factor
